# Reviewer acknowledgement 2013

**DOI:** 10.1186/2047-1440-3-3

**Published:** 2014-02-05

**Authors:** Edward K Geissler, Alan Jardine

**Affiliations:** 1 University Hospital Regensburg, Regensburg, Germany; 2 University of Glasgow and Honorary Consultant in Medicine & Nephrology at the Western Infirmary, Glasgow, UK

## Abstract

**Contributing reviewers:**

The Editors of *Transplantation Research* would like to thank all our reviewers who have contributed to the journal in Volume 2 (2013).

## 

Daniel Abramowicz

Belgium

Carla Baan

The Netherlands

Richard Baker

United Kingdom

David Briscoe

United States of America

Chris Callaghan

United Kingdom

Federica Casiraghi

Italy

Steven Chadban

Australia

Marc Clancy

United Kingdom

Edward Cole

Canada

Marc Dahlke

Germany

Jose De Lima

Brazil

Olivier De Rougemont

Switzerland

Fritz Diekmann

Spain

Philipp Dutkowski

Switzerland

Elke Eggenhofer

Germany

Stuart Flechner

United States of America

Peter Friend

United Kingdom

John Gill

Canada

Jens Goebel

United States of America

Josep Grinyó

Spain

Markus Guba

Germany

Anders Hartmann

Norway

Peter Heeger

United States of America

Hallvard Holdaas

Norway

James Hutchinson

Germany

Franz F Immer

Switzerland

Bryce Kiberd

Canada

Eiji Kobayashi

Japan

Robert Langer

Hungary

Yvon Lebranchu

France

Wai Lim

Australia

Martin Loss

Germany

Iain Macphee

United Kingdom

Patrick Mark

United Kingdom

Nicolas Meurisse

Belgium

Elmi Muller

South Africa

Björn Nashan

Germany

Ronan O'carroll

United Kingdom

Neal Padmanabhan

United Kingdom

Rajan Patel

United Kingdom

G. V. Ramesh Prasad

Canada

Lionel Rostaing

France

Birgit Sawitzki

Germany

Andreas Schnitzbauer

Germany

Antonio Secchi

Italy

Paul G Shiels

United Kingdom

Amanda Sowden

United Kingdom

R. Brian Stevens

United States of America

David Talbot

United Kingdom

Tung Yu Tsui

Germany

Gregor Warnecke

Germany

Nicholas Zavazava

United States of America

Yuan Zhai

United States of America

